# Menopausal stage differences in endothelial resistance to ischemia‐reperfusion injury

**DOI:** 10.14814/phy2.15768

**Published:** 2023-09-21

**Authors:** Jocelyn M. Delgado Spicuzza, David N. Proctor, Dick H. J. Thijssen, Yasina B. Somani

**Affiliations:** ^1^ Integrative and Biomedical Physiology, Huck Life Sciences The Pennsylvania State University University Park Pennsylvania USA; ^2^ Kinesiology Department The Pennsylvania State University University Park Pennsylvania USA; ^3^ Research Institute of Sport and Exercise Science Liverpool John Moores University Liverpool UK; ^4^ Radboud Institute of Health Sciences, Department of Physiology Radboud University Medical Center Nijmegen The Netherlands; ^5^ School of Biomedical Sciences, Faculty of Biological Sciences University of Leeds Leeds UK

## Abstract

**Background:**

In postmenopausal women, reduced ovarian function precedes endothelial dysfunction and attenuated endothelial resistance to ischemia‐reperfusion (IR) injury. We hypothesized that IR injury would lower endothelial function, with premenopausal women demonstrating the greatest protection from injury, followed by early, then late postmenopausal women.

**Methods:**

Flow‐mediated dilation (FMD) was assessed at baseline and following IR injury in premenopausal (*n* = 11), early (*n* = 11; 4 ± 1.6 years since menopause), and late (*n* = 11; 15 ± 5.5 years since menopause) postmenopausal women.

**Results:**

There were significant group differences in baseline FMD (*p* = 0.007); post hoc analysis revealed a similar resting FMD between premenopausal (7.8% ± 2.1%) and early postmenopausal (7.1% ± 2.7%), but significantly lower FMD in late postmenopausal women (4.5% ± 2.3%). Results showed an overall decline in FMD after IR injury (*p* < 0.001), and a significant condition*time interaction (*p* = 0.048), with early postmenopausal women demonstrating the most significant decline in FMD following IR.

**Conclusion:**

Our findings indicate that endothelial resistance to IR injury is attenuated in healthy early postmenopausal women.

## 1 INTRODUCTION

The onset of menopause and associated changes in ovarian hormones, particularly the loss of estrogen, often precedes endothelial dysfunction and accelerated central and peripheral arterial stiffening in women. Estrogen‐mediated maintenance of endothelial function is achieved by promoting nitric oxide (NO) synthesis via the upregulation of NO synthase, as well as other direct and indirect antioxidant and anti‐inflammatory signaling pathways (Chen, 1999; Gavin et al., 2009; Nikolic et al., 2007; Nuedling, 2001; Prorock, 2003; Simoncini et al., 2000). The absence of these protective pathways appears to play a role in menopause stage‐dependent reductions in vasodilatory capacity and increased arterial stiffening (Hildreth et al., 2014; Moreau, 2003; Samargandy et al., 2020; Zaydun et al., 2006), setting the stage for cardiovascular disease (CVD) progression (Rossi et al., 2008).

Impaired endothelial‐dependent vasodilation is predictive of CVD development in postmenopausal women, (Inaba et al., 2010; Rossi et al., 2008) and this may, in part, relate to attenuated protection against endothelial ischemia‐reperfusion (IR) injury (Prorock, 2003); a type of injury that can take place during myocardial infarction or planned surgeries and is initiated by a period of inadequate blood flow resulting in reactive oxygen species (ROS) generation followed by restoration of blood flow. Reestablishing blood flow is essential to recover the ischemic tissue; however, this can exacerbate cellular damage, due to even greater ROS formation (Kharbanda et al., 2001). Increased oxidative stress during IR may, over time, contribute to impairments in basal endothelial function in estrogen‐deficient women (Moreau et al., 2020). Activation of estrogen receptor α/β has been shown to reduce oxidative damage and improve cardioprotection against IR injury in several animal models (Booth et al., 2003; Lin et al., 2009; Wang et al., 2006). Together, the upregulation of NO and antioxidant role of estrogen is thought to sequester ROS generated during the reperfusion portion of IR injury and subsequently, reduce oxidative tissue damage to the target organ, and/or endothelium. These studies highlight the potential impact of estrogen‐mediated vascular protection from IR injury, although studies examining this in humans are currently lacking. Investigating vascular responses at discrete menopausal stages may provide additional insight into the temporal impact of estrogen deficiency on endothelial resistance to IR injury.

In the present cross‐sectional study, we compared the extent of vascular dysfunction from pneumatic cuff‐induced whole arm endothelial IR injury in estrogen‐replete premenopausal women, who served as our control group, and estrogen‐deficient women staged into early (1–6 years following menopause) and late (>6 years following menopause) postmenopausal. We hypothesized that IR injury would acutely lower endothelial function in all groups; however, estrogen‐replete premenopausal women would exhibit the greatest protection against IR injury, followed by early postmenopausal, then late postmenopausal. To account for the strong association between estrogenic status and endothelial dysfunction in women, we also included a baseline assessment of peripheral arterial stiffness.

## 2 METHODS

### 2.1 Participants

Study participants were recruited from Penn State campus and the greater surrounding State College, Pennsylvania community and provided written informed consent. Out of the 54 women screened, 33 met eligibility criteria and completed all portions of the study. All procedures were approved by the Office of Research Protections at The Pennsylvania State University in agreement with the guidelines set forth by the Declaration of Helsinki.

Participants in the premenopausal group were women in their reproductive years (with regular menstrual cycles or taking hormonal contraception). Six of the 11 premenopausal group were on hormonal contraception (3 Mirena IUD, 1 Nexplanon, 1 Camila). Of the five premenopausal women not on hormonal contraceptives, four self‐reported having regular menstrual cycles. Those in the postmenopausal group, were staged into early (1–6 years following their final menstrual cycle), and late (>6 years following their final menstrual cycle) based on the STRAW+10 criteria (Harlow et al., 2012). Menstrual cycle was not controlled for in premenopausal women. Eligible participants did not have overt chronic disease as confirmed by a physician reviewed medical history questionnaire and venous blood chemistry (hematological, liver, and kidney function), and met the following criteria: resting brachial blood pressure < 130/80 mmHg, body mass index between 18.5 and 35 kg/m^2^, fasting plasma glucose <100 mg/dL or HbA1c <6.0%, fasting plasma low‐density lipoprotein <160 mg/dL, non‐smoker, not taking any cardiovascular medications or hormone replacement therapy (postmenopausal group only), and had not donated blood or blood products in the past 3 months. Physical activity was determined using the self‐reported International Physical Activity Questionnaire (Booth, 2000). After participant eligibility was determined, volunteers were asked to visit the lab on one occasion to undergo study testing.

### 2.2 Study design

Participants arrived at the Clinical Research Center at The Pennsylvania State University between 7 and 10 a.m. having met the pretesting requirements: 12 h fasted, 12 h without caffeine, 48 h without alcohol and dietary supplements, and 24 h refraining from vigorous exercise. After voiding and 10 min of seated rest, resting blood pressure and heart rate were measured in triplicate with a 1‐min rest separating measurements. Participants rested for an additional 10 min in a supine position before pulse wave velocity (PWV; VP2000 Colin Medical) was measured in triplicate to assess arterial stiffness (Tanaka et al., 2009). After 10 min of rest, vascular assessments with Doppler ultrasound were performed before and after the IR injury protocol. All vascular assessments were performed on the same arm (right arm), in a dark, quiet, temperature‐controlled (21°C) room following expert guidelines (Thijssen et al., 2019).

### 2.3 Arterial stiffness measurements

Ankle‐brachial pulse wave velocity (PWV) was measured by placing four blood pressure cuffs securely around the participant's upper arms and ankles, ECG electrodes were placed on the inner right and left wrists, and the phonocardiogram sensor was placed on the rib cage location according to manufacturer instructions (Colin VP). The automatic measurement was initiated and lasted between 45 s and 1 min. PWV and ankle‐brachial index (ABI) were measured supine in triplicate separated by 1 min.

### 2.4 Vascular assessments

#### 2.4.1 Experimental protocol

Brachial artery flow‐mediated dilation (FMD), a method primarily mediated by NO endothelium‐dependent vasodilation, was measured in the right arm (extended 80–90 degrees from the torso). A rapid inflation/deflation pneumatic cuff (Hokanson) was placed around the forearm distal to the olecranon process. Longitudinal B‐mode images of the brachial artery in the distal portion of the upper arm were acquired using a multifrequency linear array probe attached to a high‐resolution ultrasound machine (Phillips IU22). Doppler velocity was simultaneously recorded at an insonation angle of 60 degrees and sample volume was adjusted to the vessel size. Resting brachial artery diameter and blood velocity were recorded for 1 min. The pneumatic cuff was inflated to 250 mmHg for 5 min and arterial lumen diameter and blood velocity were continuously measured during the 5‐min cuff inflation. Upon cuff deflation, imaging continued for 3 min. Transducer placement was marked on the participant's arm to minimize differences in imaging location between measurements. The same sonographer performed all FMD tests and had a coefficient of variation of 1% for baseline diameter and 16.9% for relative FMD. These values are in line with recommended expert values (Thijssen et al., 2019).

## 3 DATA ANALYSIS

Live commercial edge‐detection software was used to analyze artery diameter, blood velocity, and shear rate (FMD Studio, Cardiovascular Suite 4, Quipu, Pisa, Italy) and reviewed manually to ensure accurate wall detection. The region of interest was chosen by the same sonographer for all analyses and kept constant throughout each protocol to minimize artificial intra‐subject variation. Variables assessed using the software included baseline diameter, peak diameter, resting and peak shear rates, velocities, and shear area under the curve (SR AUC) to peak diameter. FMD was calculated as the percent increase from baseline to peak diameter during reactive hyperemia ((peak diameter‐baseline diameter)/baseline diameter * 100%).

## 4 ARTERIAL HEMODYNAMICS

Shear rate (s^−1^), the frictional force exerted by blood flow, was calculated using the following equation: 4 * mean blood velocity (cm/s)/ diameter (cm) (FMD Studio, Cardiovascular Suite 4, Quipu, Pisa, Italy). Shear rate area under the curve (SR AUC), the stimulus for FMD (Pyke & Tschakovsky, 2005), was defined as the area from the start of cuff inflation to the time of peak diameter. Blood flow is reported as a 30‐s average before cuff inflation and calculated by multiplying the cross‐sectional area (πr^2^) of the artery with resting blood velocity. Reactive hyperemia (RH) blood flow AUC and velocity AUC, indices of microvascular function, were calculated within the first minute following cuff deflation using the trapezoid method (GraphPad Prism version 8.0.0 for Windows, GraphPad Software). Peak blood flow is reported as the highest 3‐s average following cuff release and represents RH.

### 4.1 IR injury protocol

To induce a transient yet significant whole arm endothelial IR injury, a pneumatic cuff was placed around the upper portion of the right arm as close to the axilla as possible to sufficiently occlude the brachial artery. The cuff was inflated to a pressure of 250 mmHg for 20 min. The cuff was then deflated, and reperfusion occurred for 15 min before FMD was repeated. The upper arm IR injury model is a noninvasive, well‐established procedure used to study endothelial‐mediated vascular injury (Kharbanda et al., 2001).

### 4.2 Statistical analyses

Based on a previous similarly designed endothelial IR injury study in premenopausal women (Parker et al., 2016), an a priori power analysis was conducted (G*Power version 3.0.1) for an F test (ANOVA, repeated measures, within‐between interaction). Based on statistical power (1‐β) of 0.80, a medium effect size of 0.5, and an overall significance level of 0.05, at least 10 participants in each group would be sufficient to detect group differences in endothelial function before and after IR injury; our primary outcome measure.

All data were examined using descriptive statistics on SPSS software version 28 (IBM Corp.) Outliers outside of cook's distances were removed. One‐way analysis of variance (ANOVA) and post hoc tests using a Bonferroni correction were used to assess differences in participant characteristics and arterial stiffness measures. To account for the imbalanced dataset, FMD results were evaluated using a linear mixed model to assess differences in both absolute and allometrically scaled FMD (ln(peak diameter)‐ln(baseline diameter), before and after IR injury between premenopausal and postmenopausal groups. Fixed variables group and time were set to three levels: premenopausal, early and late postmenopausal; and two levels: pre‐IR and post‐IR, respectively. We used SR AUC as a covariate as this parameter can influence vascular outcomes (Atkinson & Batterham, 2013). Values in tables are reported as mean ± standard deviation.

## 5 RESULTS

### 5.1 Participant characteristics

Time since menopause in early and late postmenopausal women was 4 ± 1.6 years and 15 ± 5.5 years, respectively (*p* < 0.001). Women categorized as late postmenopausal were significantly older (63 ± 5) than early (56 ± 3, *p* < 0.001). BMI was not different across groups (*p* = 0.34); however, total cholesterol (*p* = 0.002) and LDL (*p* = 0.03) were lower in premenopausal women in comparison to postmenopausal groups (Table 1).

**TABLE 1:** Participant characteristics.

Variable	Premenopausal	Early postmenopausal	Late postmenopausal	*p*‐value
*n*	11	11	11	
Age (year)	25 ± 3 (21–30) * ^,^ **	56 ± 4 (46–60)**	63 ± 5 (57–70)	<0.001
Years since menopause		4 ± 1.6 (2–6)**	15 ± 5.5 (7–25)	<0.001
Body Mass (kg)	72 ± 12**	67 ± 11**	59 ± 5	0.01
Height (cm)	168 ± 6**	167 ± 6**	162 ± 4	0.01
BMI (kg/m^2^)	25 ± 3.4	24 ± 3.2	23 ± 2.9	0.34
Resting systolic BP (mmHg)	107 ± 8	112 ± 11	115 ± 11	0.22
Resting diastolic BP (mmHg)	70 ± 7	67 ± 9	65 ± 6	0.26
Resting HR (beats/min)^a^	66 ± 13	62 ± 6	65 ± 13	0.66
Total cholesterol (mg/dL)^a^	165 ± 34* ^,^ **	210 ± 32	215 ± 30	0.002
LDL (mg/dL)	88 ± 31* ^,^ **	123 ± 31	117 ± 30	0.03
HDL (mg/dL)	66 ± 9**	65 ± 14**	80 ± 17	0.03
Triglycerides (mg/dL)	66 ± 30	84 ± 30	83 ± 33	0.32
Fasting glucose (mg/dL)	87 ± 5	89 ± 5	92 ± 8	0.22
Hematocrit (%)	42 ± 3	41 ± 3	40 ± 3	0.61
Hemoglobin (g/dL)	14 ± 1	13 ± 1	14 ± 1	0.48
Physical activity (MET‐week)	3945 ± 3069	3064 ± 3822	2538 ± 1745	0.55
Parturition number	0	2 ± 1	2 ± 1	0.37
Pulse wave velocity (cm/s)	1072 ± 106* ^,^ **	1276 ± 192**	1434 ± 292	<0.001
Ankle brachial index	1.06 ± 0.11* ^,^ **	1.12 ± 0.04	1.12 ± 0.09	0.006

*Note*: Results of the one‐way ANOVA are represented as means ± SD.

Abbreviations: BMI, body mass index; BP, blood pressure; FMD, flow‐mediated dilation; HDL, high‐density lipoprotein; HR, heart rate; LDL, low‐density lipoprotein.

^a^ Premenopausal *n* = 10.

* *p* < 0.05, significantly different from early postmenopausal.

** *p* < 0.05 significantly different from late postmenopausal.

### 5.2 Arterial stiffness

Pairwise comparisons revealed significant differences in PWV between groups, with the highest velocity exhibited by late postmenopausal women, followed by early, then premenopausal women (*p* < 0.001, Table 1).

### 5.3 Effects of estrogen status on vascular function and protection against IR injury

**FIGURE 1:**
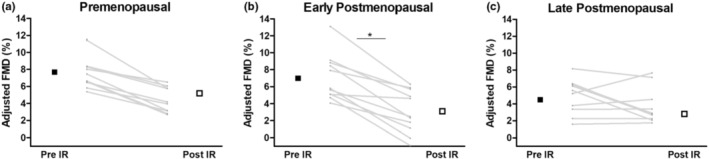
The effects of IR injury on adjusted brachial artery flow‐mediated dilation before (pre‐IR) and immediately after IR injury (post‐IR). Results of the linear mixed model with individual data points represented as gray lines and pre‐ and post‐IR group means represented as black and open squares, respectively in A. Premenopausal: pre‐IR *n* = 11, post‐IR *n* = 10; B. Early postmenopausal: pre‐ and post‐IR *n* = 11, C. Late postmenopausal: pre‐IR *n* = 11, post‐IR *n* = 10. Abbreviations; IR, ischemia reperfusion; FMD, flow‐mediated dilation; PM, postmenopausal; *p < 0.05, significantly greater decline in brachial artery FMD pre‐ and post‐IR compared to premenopausal and late postmenopausal women.

Baseline group differences in FMD were detected (*p* < 0.01); baseline FMD was similar between premenopausal and early postmenopausal, but significantly lower in late postmenopausal women (Table 2). Results of the linear mixed model using allometrically scaled FMD showed there was an overall decline in FMD after IR injury (*p* < 0.001), and a significant condition*time interaction (*p* = 0.048, Figure 1). Pairwise comparisons revealed that early postmenopausal women demonstrated the greatest decline in FMD following IR injury, while the relative decline in premenopausal and late postmenopausal women were similar.

**TABLE 2:** Effects of IR injury on brachial artery flow‐mediated dilation before (baseline) and post‐IR injury in premenopausal and postmenopausal women.

Variables	Premenopausal	Early postmenopausal	Late postmenopausal	Group	Time	Interaction
Pre‐IR						
Pre‐IR FMD (%)	7.8 ± 2.1	7.1 ± 2.7	4.5 ± 2.3	0.02	<0.001	0.052
Pre‐IR allometrically scaled FMD (%)	7.7 ± 2.3	7.0 ± 2.3*	4.5 ± 2.3	0.02	<0.001	0.048
Baseline diameter (mm)	3.4 ± 0.2	3.5 ± 0.5	3.3 ± 0.4	0.33	0.04	0.40
Peak diameter (mm)	3.6 ± 0.2	3.7 ± 0.4	3.4 ± 0.5	0.27	0.34	0.42
Time to peak (s)	45 ± 13	56 ± 16	46 ± 16	0.28	0.24	0.96
Baseline positive shear rate (s^−1^)	88.4 ± 47**	100.40 ± 47**	167.8 ± 47	0.009	0.01	0.003
Shear rate AUC (10^−3^)	17.9 ± 5.1	23.1 ± 6.6	23.8 ± 8.5	0.43	<0.001	0.16
Baseline blood flow (mL/min)	35 ± 23.6	37 ± 23.6	57 ± 23.6	0.25	0.64	0.04
Peak blood flow (mL/min)	356.3 ± 156.3	482.9 ± 156.3	414.5 ± 156.3	0.26	0.07	0.67
RH blood flow AUC (10^−3^)	19.13 ± 92.3	26.0 ± 92.3	23.9 ± 92.3	0.18	0.11	0.99
Baseline velocity (cm/s)	7.4 ± 3.7**	8.7 ± 3.7**	13.5 ± 3.7	0.01	0.53	0.01
Peak velocity (cm/s)	68.9 ± 23**	83.9 ± 23	93.6 ± 23	0.10	0.03	0.41
RH peak velocity AUC (10^−3^)	15.3 ± 5.0	20.2 ± 5.0	18.8 ± 5.0	0.11	<0.001	0.58
Post‐IR						
Post‐IR FMD (%)^a^ ^,^ ^b^	5.3 ± 2.6	3.1 ± 2.6	2.8 ± 2.8			
Post‐IR allometrically scaled FMD (%)^a^ ^,^ ^b^	5.2 ± 2.4	3.1 ± 2.4	2.8 ± 2.7			
Baseline diameter (mm)	3.4 ± 0.5	3.7 ± 0.6	3.4 ± 0.5			
Peak diameter (mm)	3.5 ± 0.5	3.8 ± 0.6	3.5 ± 0.5			
Time to peak (s)	42 ± 13	51 ± 21	43 ± 14			
Baseline positive shear rate (s^−1^)	88 ± 37	100 ± 37	111.1 ± 37			
Shear rate AUC (10^−3^)	16.5 ± 4.9	23.1 ± 6.5	15.3 ± 6.5			
Baseline blood flow (mL/min)	35 ± 22.2	49 ± 22.2	39.2 ± 22.2			
Peak blood flow (mL/min)	319.3 ± 170	401.6 ± 175	387.7 ± 175			
RH Blood flow AUC (10^−3^)	15.4 ± 12.0	21.7 ± 12.0	20.4 ± 12.0			
Baseline velocity (cm/s)	7.6 ± 2.9	9.0 ± 2.9	9.4 ± 2.9			
Peak velocity (cm/s)	66.1 ± 21.9	73.8 ± 21.9	75.5 ± 21.9			
RH peak velocity AUC (10^−3^)	12.7 ± 5.3	15.2 ± 5.3	13.4 ± 5.3			

*Note*: Results of the linear mixed model are represented as mean ± SD.

Abbreviations: AUC, area under the curve to peak; FMD, flow‐mediated dilation; RH, reactive hyperemia.

^a^ Premenopausal *n* = 10.

^b^ Late postmenopausal *n* = 10.

* *p* < 0.05, significantly greater decline in brachial artery FMD from pre‐ to post‐IR compared to premenopausal and late postmenopausal women.

** *p* < 0.05 significantly different from late postmenopausal.

### 5.4 Effects of estrogen status on hemodynamic measures following IR injury

Results of the linear mixed model revealed a significant condition*time interaction effect (*p* = 0.04) for baseline blood flow, and baseline velocity (*p* = 0.007, Table 2). Post hoc analyses revealed no significant group differences between pre‐IR and post‐IR baseline blood flow. Pairwise comparisons demonstrated that late postmenopausal women had the greatest baseline velocity compared to early postmenopausal (*p* = 0.014) and premenopausal women (*p* = 0.002).

## 6 DISCUSSION

In the present cross‐sectional study, our primary aim was to evaluate menopause‐stage differences in vascular protection against endothelial IR injury, with estrogen‐replete premenopausal women representing the control comparison. Despite comparable baseline endothelial function to premenopausal women, early postmenopausal women demonstrate an exaggerated IR‐induced impairment in endothelial function. Late postmenopausal women exhibit a post‐IR decline in endothelial function to the same level as early postmenopausal women; however, late postmenopausal women demonstrate lower baseline endothelial function prior to injury. Our secondary aim was to evaluate menopause‐stage differences in arterial stiffness; our findings reinforce previous work showing progressive arterial stiffening with reproductive aging in women (Samargandy et al., 2020; Zaydun et al., 2006).

Notably, macrovascular function declined in all groups following endothelial IR injury; with the range of this decline (premenopausal: 35%; early: 60%, late postmenopausal: 25%) comparable to other young and middle‐aged groups using a similar model of IR injury (Kharbanda et al., 2001; Lalande et al., 2021; Loukogeorgakis et al., 2005; Parker et al., 2016; Stray‐Gundersen et al., 2022). However, while both premenopausal and early postmenopausal groups demonstrated similar resting vascular function (FMD: 7.8 ± 2.1% and 7.1 ± 2.7%, respectively), IR injury induced greater endothelial dysfunction in the early postmenopausal group.

This finding reveals a potential tipping point in vascular function, highlighted by the response to IR injury. In heart failure patients, 12‐weeks of exercise training improved endothelial resistance to IR injury, without changes in resting endothelial function after the 12 weeks (Thijssen et al., 2019). Considering this sequence of events in the reverse order brings forth the idea that an initial step in the menopause‐induced decline in resting endothelial function may be, first, an attenuated protection against IR injury. Indeed, our observations show reduced endothelial resistance to IR injury in the early postmenopausal years, followed by a decline in basal endothelial function in late postmenopause; a finding that may help to explain why we did not observe an immediate decline in baseline vascular function after menopause (Holder et al., 2021). The loss of estrogen secondary to menopause may underlie our observations. The late follicular phase of the menstrual cycle, when estradiol concentrations are elevated, is associated with preservation of radial artery endothelial function in response to IR injury (Parker et al., 2016). Moreover, Hamelin and colleagues reported higher risk for acute ischemic coronary events in the early follicular phase of the menstrual cycle, when estradiol concentrations are depressed (Hamelin et al., 2003; Lloyd, 2000). This previous work suggests circulating estradiol levels are partially responsible for endothelial resistance to IR injury in premenopausal women and may be attributable to estrogen receptor alpha‐mediated increases in NO signaling (Prorock, 2003; Simoncini et al., 2000; Wang et al., 2006).

Our findings indicate that resting endothelial function following menopause may be preserved until sometime between early and late postmenopause. Previous cross‐sectional data show that macrovascular endothelial function, measured by brachial artery FMD, declines with menopausal stage (Celermajer et al., 1994; Moreau et al., 2012). However, a recent study demonstrated a minimal decline in FMD in healthy older women when strictly controlling for age and CVD risk factors (Holder et al., 2021). Interestingly, when we stratified postmenopausal women into early and late postmenopausal stages, we saw no difference in baseline FMD between early postmenopausal and premenopausal women. This may be due to the strict exclusion criteria for CVD risk factors employed in this study (Witte et al., 2005). Additionally, early postmenopausal women have significantly higher plasma L‐arginine concentrations (a substrate endogenously converted to endothelial NO) compared to perimenopausal women, which could represent a compensatory response to increase NO biosynthesis and preserve endothelial function after the sudden decline in estrogen with menopause (Klawitter et al., 2017). With more prolonged estrogen deficiency (>6 years), there is a reduction in estrogen receptor alpha function/expression that is associated with impaired endothelium‐dependent vasodilation (Gavin et al., 2009; Klawitter et al., 2017; Moreau et al., 2012; Pinna et al., 2008). Our work reinforces that prolonged estrogen deficiency in the late postmenopausal years is associated with impaired macrovascular function. The similar resting FMD between premenopausal and early postmenopausal women suggests that the early stage of the menopause represents a critical time for initiation of CVD interventions before more reductions in baseline vascular function surface.

Our observations indicate greater arterial stiffening in postmenopausal women in comparison to the premenopausal group, which may be driven, in part, by menopause‐induced changes in reproductive hormones (Gavin et al., 2012; Khan et al., 2018; Moreau, 2003; Samargandy et al., 2020; Takahashi et al., 2005; Zaydun et al., 2006). The SWAN heart study reported increased central arterial stiffness within the first year of the final menstrual period, as assessed by carotid‐femoral PWV (Samargandy et al., 2020). The notable difference in the number of years since menopause observed between early (4 ± 1.6 years) and late (15 ± 5.5 years) postmenopausal groups, and the relatively smaller difference in chronological age between groups (early: 56 ± 4 years; late: 63 ± 5 years), points to reproductive aging (i.e., prolonged estrogen deficiency) playing a lead role in menopause‐stage differences in PWV that we observed. Combined changes in reproductive hormones following menopause and chronological aging are likely both contributing to menopause‐stage differences in arterial stiffness (Gavin et al., 2009, 2012; Moreau, 2003; Samargandy et al., 2020).

### 6.1 Experimental considerations

There are some limitations in the present study. Hormone concentrations were not measured in our pre or postmenopausal participant pools and would have allowed us to confirm estrogen‐deficiency in the latter group. Menstrual cycle phase and contraceptive use were not controlled in premenopausal women and we recognize this is a limitation in our study. While recent data suggest that resting FMD may not be influenced by these factors (Shenouda et al., 2018; Stanhewicz & Wong, 1985), it is possible that differences may appear with the response to IR injury as shown previously in premenopausal women (Parker et al., 2016). Despite these limitations, our study design allowed us to interpret changes in endothelial function in response to a physiological stressor, that is relevant to CVD development, in two discrete menopausal stages. However, endothelial dysregulation cannot solely be accredited to changes in sex hormone concentrations; additional factors including chronological age, physical fitness, and oxidative stress status should be considered. Future longitudinal studies are required to confirm that estrogen and/or other hormones (i.e., follicle‐stimulating hormone) mediates endothelial resistance to IR injury in women across the lifespan.

## 7 CONCLUSIONS AND POTENTIAL CLINICAL RELEVANCE

We demonstrate for the first time that endothelial resistance to vascular IR injury is diminished in the early postmenopausal years in healthy women. The finding that protection against IR is attenuated to a greater extent in early postmenopausal women than premenopausal women, despite demonstrating similar baseline vascular function, has implications for CVD prevention strategies. First, our observation highlights that, even in the presence of preserved endothelial function, relevant adaptations are present that affect vascular resistance during early menopause. Second, work by Mehta and colleagues supports that this early period of menopause is the most clinically relevant stage for initiation of CVD interventions such as short‐term hormone therapy and statin treatment (Mehta et al., 2019). Overall, our results emphasize the importance of CVD interventions in the 6‐year window following the final menstrual period.

## FUNDING INFORMATION

HHS | NIH | National Center for Advancing Translational Sciences (NCATS) UL1TR002014.

HHS | NIH | National Institute of General Medical Sciences (NIGMS) T32GM108563.

HHS | NIH | National Institute of Diabetes and Digestive and Kidney Diseases (NIDDK) T32DK120509.

Gouvernement du Canada | Canadian Institutes of Health Research (IRSC) MFE‐171178.

## CONFLICT OF INTEREST STATEMENT

The authors have no conflicts of interest to declare.

## ETHICS STATEMENT

All subjects gave their informed consent for inclusion before they participated in the study. The study was conducted in accordance with the Declaration of Helsinki, and the protocol was approved by the Ethics Committee of The Pennsylvania State University (Study#10017).
